# Supplementary material for: Dysbindin Domain-Containing 1 in Prostate Cancer: New Insights into Bioinformatic Validation of Molecular and Immunological Features
Source: Int J Mol Sci. 2023 Jul 25;24(15):11930. doi: 10.3390/ijms241511930 (PMC10418609; doi:10.3390/ijms241511930)
Supplement: Supplementary file 1 [file ijms-24-11930-s001.zip › ijms-2477804-supplementary.pdf]

# THE SUPPLEMENTARY TABLES & FIGURES

## Supplementary Table S1

**Supplementary Table S1. The top 273 genes for the MetaCore analysis extracted from TCGA**

| No. | Correlated Gene | Spearman's Correlation | p-Value |
|---|---|---|---|
| 1 | APRT | 0.669646244 | 8.73E-65 |
| 2 | SLC25A10 | 0.585874288 | 2.61E-46 |
| 3 | ACY1 | 0.574263342 | 3.70E-44 |
| 4 | SLC25A39 | 0.561980496 | 5.66E-42 |
| 5 | PPP1R14B | 0.558187831 | 2.56E-41 |
| 6 | PYCR1 | 0.542233971 | 1.20E-38 |
| 7 | POLD2 | 0.5406823 | 2.14E-38 |
| 8 | SIGMAR1 | 0.53733003 | 7.44E-38 |
| 9 | CGREF1 | 0.536231365 | 1.11E-37 |
| 10 | PPIA | 0.535028926 | 1.73E-37 |
| 11 | RAC3 | 0.530840369 | 7.94E-37 |
| 12 | SPATA33 | 0.525009456 | 6.39E-36 |
| 13 | MRPL12 | 0.518974919 | 5.30E-35 |
| 14 | CTU2 | 0.515248621 | 1.92E-34 |
| 15 | PAFAH1B3 | 0.508719413 | 1.76E-33 |
| 16 | SAMD10 | 0.506656538 | 3.50E-33 |
| 17 | KATNB1 | 0.504363889 | 7.49E-33 |
| 18 | HMG20B | 0.503243849 | 1.08E-32 |
| 19 | GAS8 | 0.502603921 | 1.34E-32 |
| 20 | POLDIP2 | 0.50159287 | 1.86E-32 |
| 21 | BSG | 0.500848058 | 2.38E-32 |
| 22 | PPM1G | 0.497974698 | 6.06E-32 |
| 23 | ALG3 | 0.496320092 | 1.03E-31 |
| 24 | GGCT | 0.496293452 | 1.04E-31 |
| 25 | MFSD12 | 0.493107633 | 2.89E-31 |
| 26 | HMBS | 0.492358394 | 3.67E-31 |
| 27 | SRM | 0.489118262 | 1.02E-30 |
| 28 | RITA1 | 0.487664596 | 1.61E-30 |
| 29 | DDX49 | 0.487434033 | 1.73E-30 |
| 30 | GNB2 | 0.486549719 | 2.29E-30 |
| 31 | NMRAL1 | 0.485659739 | 3.02E-30 |
| 32 | NALF2 | 0.484133276 | 4.84E-30 |
| 33 | PGLS | 0.483082213 | 6.70E-30 |
| 34 | TIMM13 | 0.482939472 | 7.00E-30 |
| 35 | NME1 | 0.478780589 | 2.50E-29 |

| | | | |
|---|---|---|---|
| 36 | ACOT8 | 0.477897217 | 3.27E-29 |
| 37 | SRD5A1 | 0.477878941 | 3.29E-29 |
| 38 | SLC4A5 | 0.477257598 | 3.97E-29 |
| 39 | FARSA | 0.475614375 | 6.51E-29 |
| 40 | NPRL2 | 0.475219222 | 7.33E-29 |
| 41 | DDX41 | 0.473027842 | 1.41E-28 |
| 42 | HSD17B10 | 0.473027635 | 1.41E-28 |
| 43 | ASPHD1 | 0.472892716 | 1.47E-28 |
| 44 | AP1M2 | 0.472646343 | 1.58E-28 |
| 45 | UQCC3 | 0.472373948 | 1.72E-28 |
| 46 | ZNF524 | 0.472325016 | 1.74E-28 |
| 47 | SLC27A5 | 0.471544684 | 2.20E-28 |
| 48 | NUTF2 | 0.471470556 | 2.25E-28 |
| 49 | PKP3 | 0.471282099 | 2.37E-28 |
| 50 | SAPCD2 | 0.47083272 | 2.71E-28 |
| 51 | STK32C | 0.47046729 | 3.02E-28 |
| 52 | DNPH1 | 0.469302643 | 4.26E-28 |
| 53 | DTYMK | 0.468654724 | 5.16E-28 |
| 54 | C19ORF54 | 0.468553686 | 5.31E-28 |
| 55 | FIBP | 0.466609183 | 9.39E-28 |
| 56 | RABGGTA | 0.466551334 | 9.55E-28 |
| 57 | SLC19A1 | 0.466114243 | 1.09E-27 |
| 58 | B3GAT3 | 0.46521435 | 1.41E-27 |
| 59 | CHMP1A | 0.464441154 | 1.76E-27 |
| 60 | CENPX | 0.464351526 | 1.81E-27 |
| 61 | TKFC | 0.46416015 | 1.91E-27 |
| 62 | SSC4D | 0.463745505 | 2.16E-27 |
| 63 | DHPS | 0.463473548 | 2.34E-27 |
| 64 | MAZ | 0.462664704 | 2.95E-27 |
| 65 | CENPBD1P | 0.462362468 | 3.22E-27 |
| 66 | POP7 | 0.461627478 | 3.98E-27 |
| 67 | ZP3 | 0.461409707 | 4.23E-27 |
| 68 | AP1S1 | 0.461281679 | 4.39E-27 |
| 69 | KARS1 | 0.460913346 | 4.88E-27 |
| 70 | YIF1A | 0.460313171 | 5.79E-27 |
| 71 | RUVBL2 | 0.459960171 | 6.41E-27 |
| 72 | PRR19 | 0.459667435 | 6.97E-27 |
| 73 | CENPM | 0.459271647 | 7.80E-27 |
| 74 | MCRIP2 | 0.458891658 | 8.69E-27 |
| 75 | HYAL3 | 0.457926196 | 1.14E-26 |
| 76 | PRELID1 | 0.457920012 | 1.15E-26 |
| 77 | MLST8 | 0.457192754 | 1.41E-26 |

| | | | |
|---|---|---|---|
| 78 | MRTO4 | 0.456809679 | 1.57E-26 |
| 79 | DEAF1 | 0.455771925 | 2.10E-26 |
| 80 | PGAP2 | 0.455697785 | 2.15E-26 |
| 81 | HGH1 | 0.455097857 | 2.54E-26 |
| 82 | NHP2 | 0.454806153 | 2.76E-26 |
| 83 | TOMM40 | 0.454505529 | 3.00E-26 |
| 84 | SLC2A4RG | 0.454244533 | 3.23E-26 |
| 85 | IDH3G | 0.453905124 | 3.55E-26 |
| 86 | NELFCD | 0.453834082 | 3.62E-26 |
| 87 | DUS1L | 0.453796393 | 3.66E-26 |
| 88 | MPST | 0.452852202 | 4.77E-26 |
| 89 | PYCR3 | 0.452768253 | 4.88E-26 |
| 90 | TRAPPC2L | 0.452392974 | 5.42E-26 |
| 91 | SPDEF | 0.451988346 | 6.07E-26 |
| 92 | ECE2 | 0.451731039 | 6.51E-26 |
| 93 | AKT1 | 0.451627356 | 6.71E-26 |
| 94 | PLEKHJ1 | 0.451275659 | 7.39E-26 |
| 95 | ECHS1 | 0.451047729 | 7.88E-26 |
| 96 | LRFN1 | 0.451013281 | 7.95E-26 |
| 97 | ELMO3 | 0.450743468 | 8.57E-26 |
| 98 | NCLN | 0.450368085 | 9.51E-26 |
| 99 | EIF3B | 0.449416563 | 1.24E-25 |
| 100 | MRPS2 | 0.449175312 | 1.32E-25 |
| 101 | MON1A | 0.448254033 | 1.70E-25 |
| 102 | ZNF511 | 0.448126085 | 1.76E-25 |
| 103 | AKT1S1 | 0.448010332 | 1.82E-25 |
| 104 | PRMT7 | 0.4478445 | 1.91E-25 |
| 105 | EXOSC5 | 0.447732465 | 1.97E-25 |
| 106 | ATP6V1F | 0.447617189 | 2.03E-25 |
| 107 | EEF2KMT | 0.447370156 | 2.17E-25 |
| 108 | RUVBL1 | 0.447095478 | 2.34E-25 |
| 109 | CDT1 | 0.447025664 | 2.39E-25 |
| 110 | PRR7 | 0.447015648 | 2.39E-25 |
| 111 | PODXL2 | 0.446003732 | 3.15E-25 |
| 112 | CPNE7 | 0.445528332 | 3.59E-25 |
| 113 | DPM2 | 0.444798596 | 4.38E-25 |
| 114 | MCAT | 0.444672323 | 4.53E-25 |
| 115 | MRPS24 | 0.444597966 | 4.62E-25 |
| 116 | ESRP2 | 0.444147555 | 5.23E-25 |
| 117 | RPUSD1 | 0.442613245 | 7.91E-25 |
| 118 | SYNGR2 | 0.441233305 | 1.15E-24 |
| 119 | LSM4 | 0.440830289 | 1.28E-24 |

| | | | |
|---|---|---|---|
| 120 | BDH1 | 0.440436165 | 1.42E-24 |
| 121 | TCF25 | 0.440254832 | 1.49E-24 |
| 122 | BCS1L | 0.440127206 | 1.54E-24 |
| 123 | MRPS15 | 0.439359792 | 1.89E-24 |
| 124 | TRUB2 | 0.43923485 | 1.96E-24 |
| 125 | NR2F6 | 0.438713873 | 2.25E-24 |
| 126 | MOSPD3 | 0.438459796 | 2.41E-24 |
| 127 | FMC1 | 0.438054715 | 2.68E-24 |
| 128 | PCBD1 | 0.437084193 | 3.47E-24 |
| 129 | DPCD | 0.436926725 | 3.62E-24 |
| 130 | MRPL48 | 0.435359991 | 5.47E-24 |
| 131 | LAGE3 | 0.435180942 | 5.73E-24 |
| 132 | BPHL | 0.435048772 | 5.93E-24 |
| 133 | MARCHF9 | 0.434954704 | 6.08E-24 |
| 134 | RFXANK | 0.434876538 | 6.21E-24 |
| 135 | MRPL15 | 0.434644322 | 6.60E-24 |
| 136 | PAM16 | 0.434567693 | 6.73E-24 |
| 137 | YIF1B | 0.434314412 | 7.20E-24 |
| 138 | METTL26 | 0.434140618 | 7.53E-24 |
| 139 | TBRG4 | 0.433835812 | 8.16E-24 |
| 140 | RPL13 | 0.43382248 | 8.19E-24 |
| 141 | RRP9 | 0.433321782 | 9.33E-24 |
| 142 | MRPS34 | 0.432700582 | 1.10E-23 |
| 143 | COA4 | 0.432617253 | 1.12E-23 |
| 144 | BTBD2 | 0.432487768 | 1.16E-23 |
| 145 | RAD23A | 0.431947419 | 1.34E-23 |
| 146 | CORO1B | 0.430542697 | 1.92E-23 |
| 147 | FAAP100 | 0.430408255 | 1.99E-23 |
| 148 | POLR2H | 0.429964039 | 2.24E-23 |
| 149 | ZMYND19 | 0.429813283 | 2.32E-23 |
| 150 | DANCR | 0.429764648 | 2.35E-23 |
| 151 | EIPR1 | 0.429512079 | 2.51E-23 |
| 152 | CLPP | 0.427471466 | 4.25E-23 |
| 153 | IFI27L1 | 0.427463153 | 4.26E-23 |
| 154 | CDK16 | 0.427359254 | 4.37E-23 |
| 155 | CBS | 0.427065072 | 4.71E-23 |
| 156 | NDUFA7 | 0.426957477 | 4.85E-23 |
| 157 | PDZD11 | 0.426525881 | 5.41E-23 |
| 158 | CACFD1 | 0.42589455 | 6.36E-23 |
| 159 | AP2S1 | 0.42558901 | 6.87E-23 |
| 160 | TPRN | 0.425064655 | 7.86E-23 |
| 161 | YBEY | 0.424738566 | 8.54E-23 |

| | | | |
|---|---|---|---|
| 162 | EMC10 | 0.424729067 | 8.56E-23 |
| 163 | NAA10 | 0.424649248 | 8.73E-23 |
| 164 | SPATA2L | 0.424328189 | 9.47E-23 |
| 165 | MARVELD3 | 0.423927681 | 1.05E-22 |
| 166 | TALDO1 | 0.42375111 | 1.10E-22 |
| 167 | ATAD3A | 0.423545627 | 1.16E-22 |
| 168 | SNRPA | 0.422887254 | 1.36E-22 |
| 169 | COX5A | 0.422812764 | 1.39E-22 |
| 170 | KRT18 | 0.422755394 | 1.41E-22 |
| 171 | PEBP1 | 0.422175353 | 1.63E-22 |
| 172 | MTX1 | 0.420919054 | 2.24E-22 |
| 173 | CINP | 0.420715326 | 2.36E-22 |
| 174 | CDK10 | 0.420677533 | 2.38E-22 |
| 175 | NDUFAF3 | 0.420509739 | 2.48E-22 |
| 176 | RNASEH2A | 0.420437624 | 2.53E-22 |
| 177 | SYNE4 | 0.420295995 | 2.62E-22 |
| 178 | CAMK2B | 0.419009917 | 3.61E-22 |
| 179 | TIMM50 | 0.418319638 | 4.29E-22 |
| 180 | GTF3A | 0.418026654 | 4.61E-22 |
| 181 | MFSD3 | 0.41796918 | 4.68E-22 |
| 182 | NCBP2AS2 | 0.416980382 | 5.98E-22 |
| 183 | LBHD1 | 0.416779442 | 6.28E-22 |
| 184 | NOP16 | 0.41676323 | 6.31E-22 |
| 185 | DHODH | 0.416725448 | 6.37E-22 |
| 186 | SMARCD2 | 0.416077494 | 7.47E-22 |
| 187 | CLN6 | 0.416073983 | 7.48E-22 |
| 188 | KDF1 | 0.41598198 | 7.65E-22 |
| 189 | MRPL41 | 0.415901852 | 7.80E-22 |
| 190 | MRPS33 | 0.415875274 | 7.86E-22 |
| 191 | RPP25L | 0.415787401 | 8.03E-22 |
| 192 | P4HTM | 0.415733615 | 8.14E-22 |
| 193 | NME2 | 0.415649283 | 8.31E-22 |
| 194 | ATP5MC1 | 0.415574421 | 8.46E-22 |
| 195 | SGTA | 0.415560791 | 8.49E-22 |
| 196 | KCTD13 | 0.415316906 | 9.02E-22 |
| 197 | MRPS12 | 0.414827763 | 1.02E-21 |
| 198 | CFAP65 | 0.414392633 | 1.13E-21 |
| 199 | TMEM161A | 0.414333054 | 1.15E-21 |
| 200 | NKIRAS2 | 0.414169596 | 1.20E-21 |
| 201 | ALKBH2 | 0.414103408 | 1.21E-21 |
| 202 | MRPL57 | 0.414090976 | 1.22E-21 |
| 203 | NDUFB10 | 0.41394151 | 1.26E-21 |

| | | | |
|---|---|---|---|
| 204 | CDKN2AIPNL | 0.413867567 | 1.29E-21 |
| 205 | FAM174C | 0.41358051 | 1.38E-21 |
| 206 | FAM89B | 0.413466822 | 1.42E-21 |
| 207 | DHRS11 | 0.413278501 | 1.49E-21 |
| 208 | ERAL1 | 0.413059377 | 1.57E-21 |
| 209 | TUFM | 0.412887721 | 1.64E-21 |
| 210 | EIF3I | 0.412558628 | 1.77E-21 |
| 211 | TIMM17B | 0.412301319 | 1.89E-21 |
| 212 | GLOD5 | 0.412076619 | 1.99E-21 |
| 213 | THEM6 | 0.411609212 | 2.23E-21 |
| 214 | HSPBP1 | 0.411580723 | 2.25E-21 |
| 215 | SNRNP25 | 0.411414054 | 2.34E-21 |
| 216 | MRPL37 | 0.411318953 | 2.40E-21 |
| 217 | PRELID1P6 | 0.411055129 | 2.56E-21 |
| 218 | DESI1 | 0.411023388 | 2.58E-21 |
| 219 | TTLL1 | 0.410748763 | 2.75E-21 |
| 220 | NFS1 | 0.410324269 | 3.05E-21 |
| 221 | CSKMT | 0.410229272 | 3.12E-21 |
| 222 | SLC29A2 | 0.410181392 | 3.16E-21 |
| 223 | UBE2M | 0.410070255 | 3.25E-21 |
| 224 | ATPAF2 | 0.410038049 | 3.27E-21 |
| 225 | TRPT1 | 0.409882945 | 3.40E-21 |
| 226 | COPE | 0.409674127 | 3.57E-21 |
| 227 | EPN1 | 0.409334449 | 3.88E-21 |
| 228 | ADM2 | 0.409247185 | 3.96E-21 |
| 229 | RNF187 | 0.409240691 | 3.97E-21 |
| 230 | TMEM208 | 0.408320445 | 4.95E-21 |
| 231 | DVL1 | 0.408219056 | 5.07E-21 |
| 232 | DCXR | 0.407801636 | 5.61E-21 |
| 233 | RALY | 0.407792147 | 5.62E-21 |
| 234 | ANAPC11 | 0.407767726 | 5.65E-21 |
| 235 | TREX1 | 0.407720206 | 5.72E-21 |
| 236 | GPS1 | 0.407452872 | 6.10E-21 |
| 237 | NDOR1 | 0.406824857 | 7.09E-21 |
| 238 | SLIRP | 0.406740815 | 7.23E-21 |
| 239 | BOLA3 | 0.406421118 | 7.80E-21 |
| 240 | TAF10 | 0.406071445 | 8.48E-21 |
| 241 | TTLL12 | 0.406018825 | 8.59E-21 |
| 242 | TXN | 0.405945408 | 8.74E-21 |
| 243 | TRPM4 | 0.405902247 | 8.83E-21 |
| 244 | PDF | 0.405870237 | 8.90E-21 |
| 245 | PDXP | 0.40584563 | 8.95E-21 |

| | | | |
|---|---|---|---|
| 246 | OXLD1 | 0.405813548 | 9.02E-21 |
| 247 | TUBB4A | 0.405673737 | 9.33E-21 |
| 248 | GATD3 | 0.405582973 | 9.53E-21 |
| 249 | OAZ1 | 0.405548072 | 9.61E-21 |
| 250 | GARIN5A | 0.405338355 | 1.01E-20 |
| 251 | NOP10 | 0.404771365 | 1.16E-20 |
| 252 | BOLA2 | 0.404765996 | 1.16E-20 |
| 253 | ASPSCR1 | 0.404573213 | 1.21E-20 |
| 254 | ITPA | 0.404334377 | 1.28E-20 |
| 255 | C2CD2L | 0.404017995 | 1.38E-20 |
| 256 | H2AC17 | 0.402944277 | 1.78E-20 |
| 257 | ELL3 | 0.402862641 | 1.82E-20 |
| 258 | CDK5 | 0.402722933 | 1.88E-20 |
| 259 | MYL5 | 0.402705689 | 1.89E-20 |
| 260 | ZNF821 | 0.402424828 | 2.02E-20 |
| 261 | FKBP4 | 0.402336253 | 2.06E-20 |
| 262 | C19ORF73 | 0.40228068 | 2.09E-20 |
| 263 | RAB1B | 0.402043496 | 2.20E-20 |
| 264 | USE1 | 0.402025426 | 2.21E-20 |
| 265 | RANGAP1 | 0.40185536 | 2.30E-20 |
| 266 | PDCD2L | 0.401407334 | 2.56E-20 |
| 267 | PRDX4 | 0.401057795 | 2.78E-20 |
| 268 | SOX12 | 0.400517725 | 3.16E-20 |
| 269 | MICOS13 | 0.400486366 | 3.18E-20 |
| 270 | MRPS17 | 0.400332718 | 3.29E-20 |
| 271 | PPP1CA | 0.400136734 | 3.45E-20 |
| 272 | GLRX5 | 0.400067758 | 3.51E-20 |
| 273 | WBP1 | 0.400021808 | 3.54E-20 |

## Supplementary Table S2

**Supplementary Table S2. Pathway maps and network objects in the MetaCore analysis.**

| No. | Enrichment of pathway maps | p-Value | Network Objects from Active Data |
|---|---|---|---|
| 1 | Regulation of GSK3 beta in bipolar disorder | 3.640E-05 | AKT1, DVL-1, AKT(PKB), Dsh, PP1-cat |
| 2 | ATP/ITP metabolism | 6.907E-05 | ITPA, APRT, NDPK A, NDPK B, RPB8, NDPK complex, RRP46 |
| 3 | GTP-XTP metabolism | 9.711E-05 | ITPA, NDPK A, NDPK B, RPB8, NDPK complex, RRP46 |
| 4 | TTP metabolism | 2.397E-04 | NDPK A, NDPK B, NDPK complex, Thymidylate kinase, POLD reg (p50) |
| 5 | CTP/UTP metabolism | 2.518E-04 | ITPA, NDPK A, NDPK B, RPB8, NDPK complex, RRP46 |
| 6 | Role of activation of WNT signaling in the progression of lung cancer | 4.285E-04 | DVL-1, ARD1, CD147, Dsh, RUVBL1 |
| 7 | Inhibition of remyelination in multiple sclerosis: regulation of cytoskeleton proteins | 4.607E-04 | Tubulin beta, CDK5, MRLC, Tubulin beta 4 |
| 8 | GTP metabolism | 1.008E-03 | ITPA, NDPK A, NDPK B, NDPK complex |
| 9 | dATP/dITP metabolism | 1.066E-03 | ITPA, NDPK A, NDPK B, NDPK complex, POLD reg (p50) |
| 10 | Development_Regulation of cytoskeleton proteins in oligodendrocyte differentiation and myelination | 1.405E-03 | Tubulin beta, CDK5, MRLC, Tubulin beta 4 |
| 11 | Neuroprotective action of lithium | 1.793E-03 | CDK5, Thioredoxin, Dsh, PP1-cat |
| 12 | dGTP metabolism | 1.901E-03 | NDPK A, NDPK B, NDPK complex, POLD reg (p50) |
| 13 | Deregulation of canonical WNT signaling in major depressive disorder | 2.073E-03 | AKT1, DVL-1, Dsh |
| 14 | G-protein signaling_Rac1 inhibition | 2.073E-03 | CDK5, AKT1, NDPK A |
| 15 | N-Glycan biosynthesis p1 | 2.927E-03 | DPM2 (reg), ALG10-A, ALG3, OST48 |
| 16 | Signal transduction_mTORC1 upstream signaling | 3.233E-03 | PRAS40, GBL, AKT(PKB), Dsh |
| 17 | dCTP/dUTP metabolism | 3.233E-03 | NDPK A, NDPK B, NDPK complex, POLD reg (p50) |
| 18 | Translation_Regulation of EIF2 activity | 3.731E-03 | PP1-cat alpha, AKT(PKB), PP1-cat |
| 19 | Butanoic acid metabolism | 4.018E-03 | ECHS1, PTE1, HCD2 |
| 20 | CHDI_DEGs from Replication data_Causal network | 4.473E-03 | CDK5, AKT(PKB), Dsh, PP1-cat |
| 21 | Apoptosis and survival_BAD phosphorylation | 4.958E-03 | PP1-cat alpha, PP2C, AKT(PKB) |

| | | | |
|---|---|---|---|
| 22 | Development_Role of CDK5 in the nervous system | 5.764E-03 | Tubulin beta, CDK5, PCTK1, AKT(PKB) |
| 23 | Neurophysiological process_HTR2A signaling in the nervous system | 7.633E-03 | SFK, AKT(PKB), SRD5A1 |
| 24 | Development_NOTCH signaling inhibition | 8.429E-03 | Presenilin 2, AKT1, RITA1, AKT(PKB) |
| 25 | LRRK2 in neuronal apoptosis in Parkinson's disease | 8.454E-03 | AKT1, Thioredoxin |
| 26 | Transport_RAN regulation pathway | 9.460E-03 | NTF2, RanGAP1 |
| 27 | ATP metabolism | 1.036E-02 | ITPA, NDPK A, NDPK B, NDPK complex |
| 28 | Aberrant B-Raf signaling in melanoma progression | 1.049E-02 | AKT1, RKIP, AKT(PKB) |
| 29 | Cytoskeleton remodeling_Alpha-1A adrenergic receptor-dependent inhibition of PI3K | 1.052E-02 | MRLC, AKT(PKB) |
| 30 | Cell adhesion_Desmosomes | 1.052E-02 | Plakophilin 3, Keratin 18 |
| 31 | Cytoskeleton remodeling_Regulation of actin cytoskeleton organization by the kinase effectors of Rho GTPases | 1.212E-02 | Rac3, MRLC, Rac1-related |
| 32 | Development_H3K9 demethylases in pluripotency maintenance of stem cells | 1.398E-02 | AKT1, AKT(PKB) |
| 33 | Inhibition of GSK3 beta by lithium in major depressive disorder | 1.398E-02 | AKT(PKB), PP1-cat |
| 34 | Stem cells_Aberrant Wnt signaling in medulloblastoma stem cells | 1.523E-02 | AKT(PKB), Dsh |
| 35 | Canonical WNT signaling pathway in colorectal cancer | 1.714E-02 | AKT1, DVL-1, Dsh |
| 36 | Development_ErbB4 signaling | 1.784E-02 | CDK5, SFK, AKT(PKB) |
| 37 | Ligand-independent activation of Androgen receptor in Prostate Cancer | 1.784E-02 | NCOA3 (pCIP/SRC3), AKT(PKB), SRD5A1 |
| 38 | Apoptosis and survival_Role of nuclear PI3K in NGF/ TrkA signaling | 1.787E-02 | AKT1, AKT(PKB) |
| 39 | Signal transduction_mTORC2 downstream signaling | 1.856E-02 | AKT1, PRAS40, AKT(PKB) |
| 40 | Nicotine / nAChR alpha-3/nAChR beta-2 signaling in NSCLC | 1.925E-02 | AKT1, AKT(PKB) |
| 41 | Transcription_Negative regulation of HIF1A function | 1.929E-02 | PRDX4, ARD1, RUVBL2 |
| 42 | Signal transduction_Ephrin reverse signaling | 2.216E-02 | MRLC, SFK |
| 43 | CHDI_Correlations from Discovery data_Causal network | 2.236E-02 | CDK5, AKT(PKB), Dsh |
| 44 | Development_Positive regulation of WNT/Beta-catenin signaling in the cytoplasm | 2.483E-02 | AKT(PKB), Dsh, PP1-cat |
| 45 | DNA damage_Intra S-phase checkpoint | 2.483E-02 | Cdt1, PP1-cat alpha, PP1-cat |
| 46 | Apoptosis and survival_NGF/ TrkA PI3K-mediated signaling | 2.569E-02 | AKT1, MRLC, AKT(PKB) |

| | | | |
|---|---|---|---|
| 47 | Role of inhibition of WNT signaling in the progression of lung cancer | 2.683E-02 | Keratin 18, Dsh |
| 48 | Immune response_IL-23 signaling pathway | 2.683E-02 | AKT1, AKT(PKB) |
| 49 | NETosis in SLE | 2.683E-02 | Histone H2, Histone H2A |
| 50 | Development_Androgen receptor in non-reproductive systems development | 2.835E-02 | NCOA3 (pCIP/SRC3), AKT(PKB), SRD5A1 |

## Supplementary Figure S1

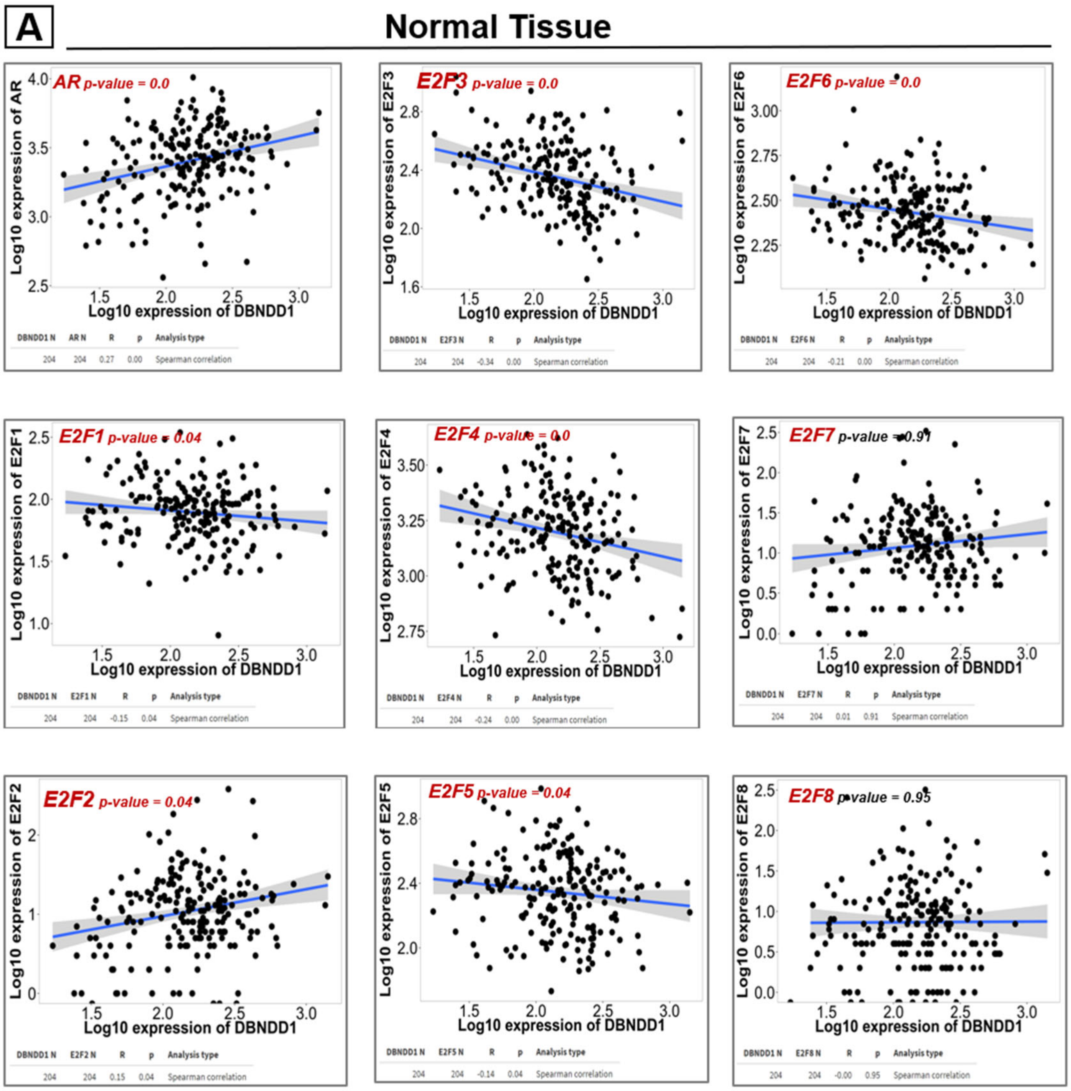

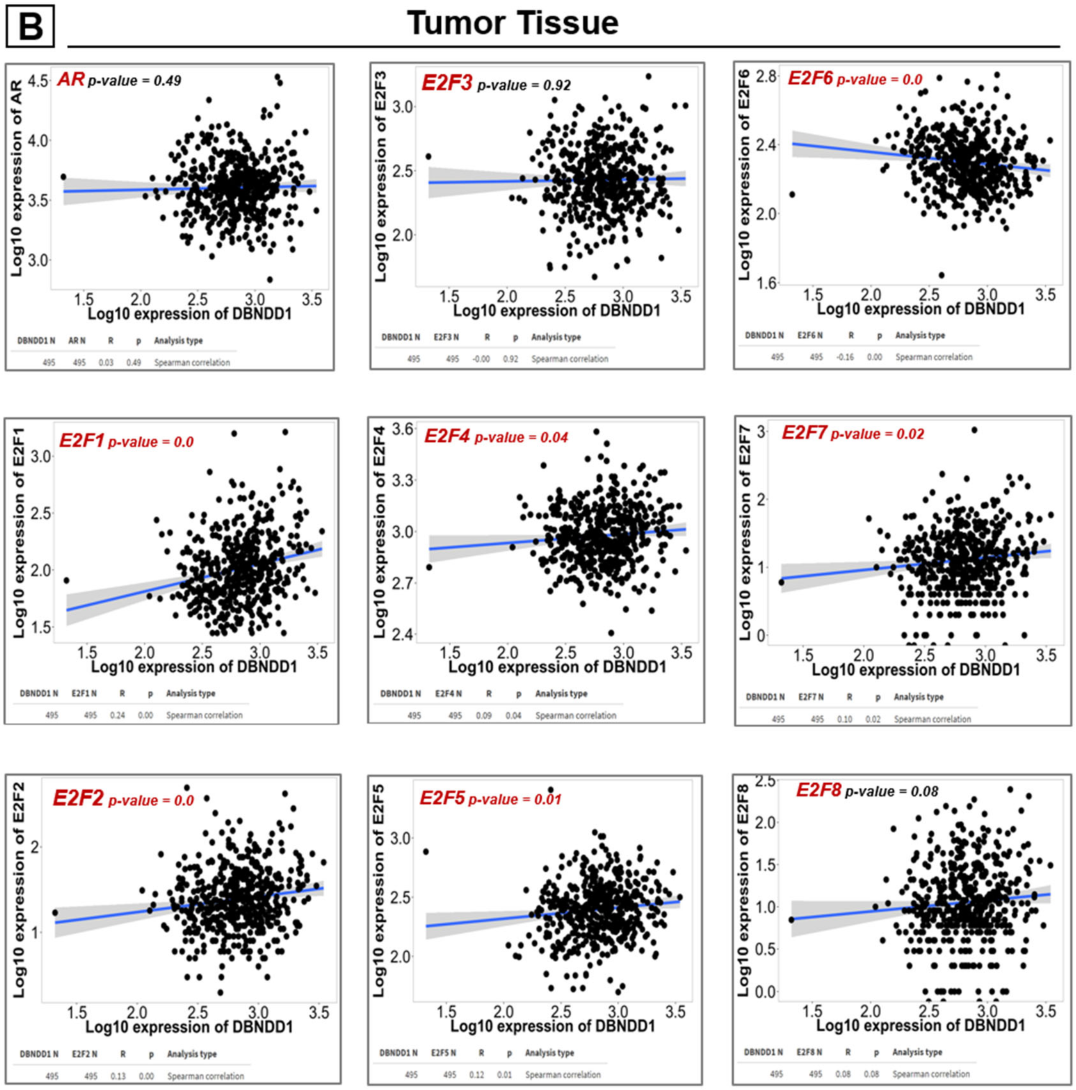


**Supplementary Figure S1. Interactions of DBNDD1 expression with the androgen receptor (AR) and E2Fs in prostate cancer using RNA-seq data.** The correlations between the genes were described using Spearman correlations ($p < 0.05$ was considered statistically significant).

## Supplementary Figure S2

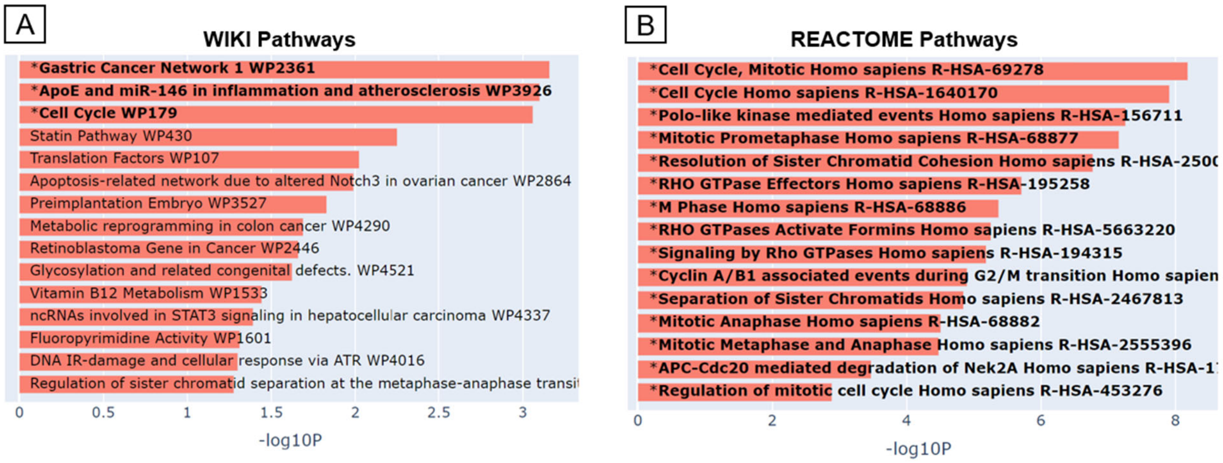


**Supplementary Figure S2. Gene ontology (GO) enrichment results for primary tumor tissues vs. solid normal tissues in TCGA prostate cancer differential gene expression analysis.** Results of an enrichment analysis including WIKI pathways (**A)**, and Reactome pathways (**B**). Important phrases are emphasized in boldface font.

## Supplementary Figure S3

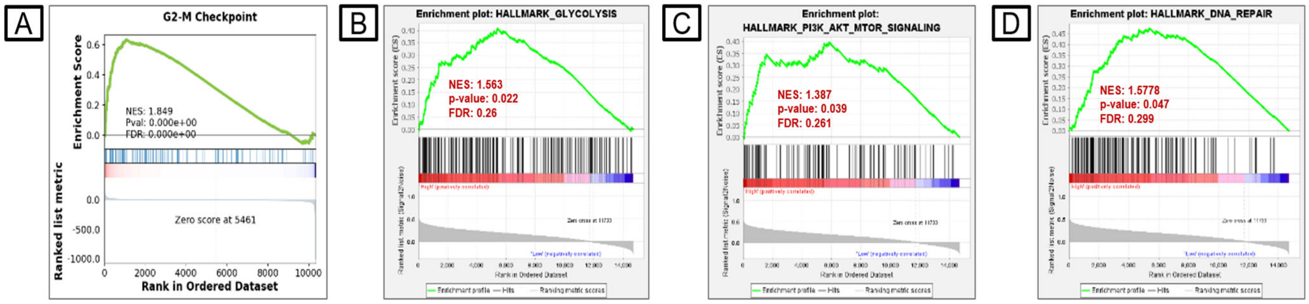


**Supplementary Figure S3. Results of a gene set enrichment analysis (GSEA) in prostate cancer patients in TCGA cohort with high *DBNDD1* expression.** (**A-D**) Patients were separated into two groups based on their *DBNDD1* mRNA expression levels in TCGA PRAD dataset; afterward, a gene ranking list was produced and entered into the GSEA. As indicated by the GSEA database, statistical significance was evaluated using a false detection rate (FDR) value of 0.25, a normalized enrichment score (NES) of more than 1.3,

and a nominal *p*-value of 0.05. Enrichment at the top of the list is indicated by a positive NES value, which suggests the enrichment pathway.

**Supplementary Figure S4**

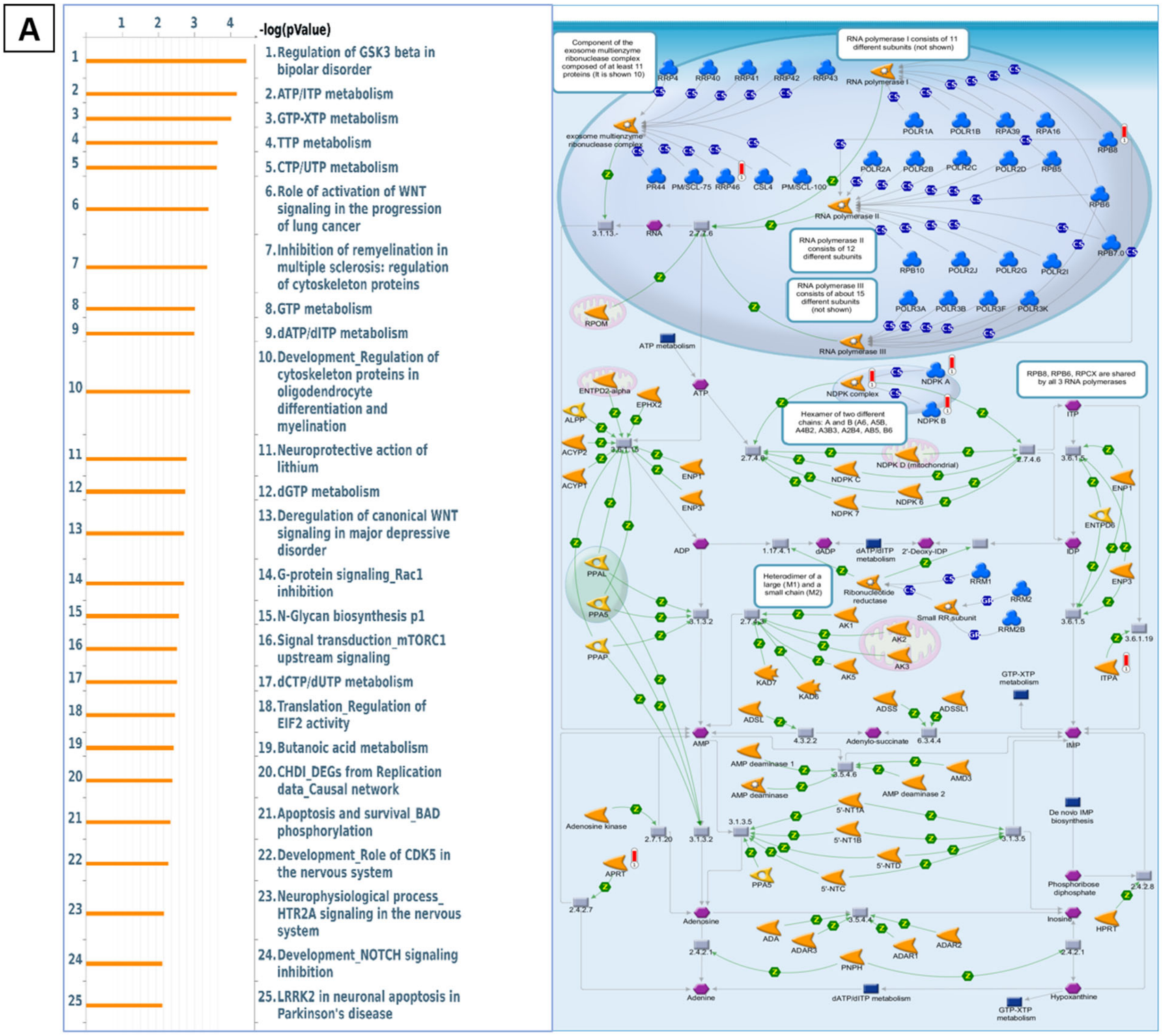

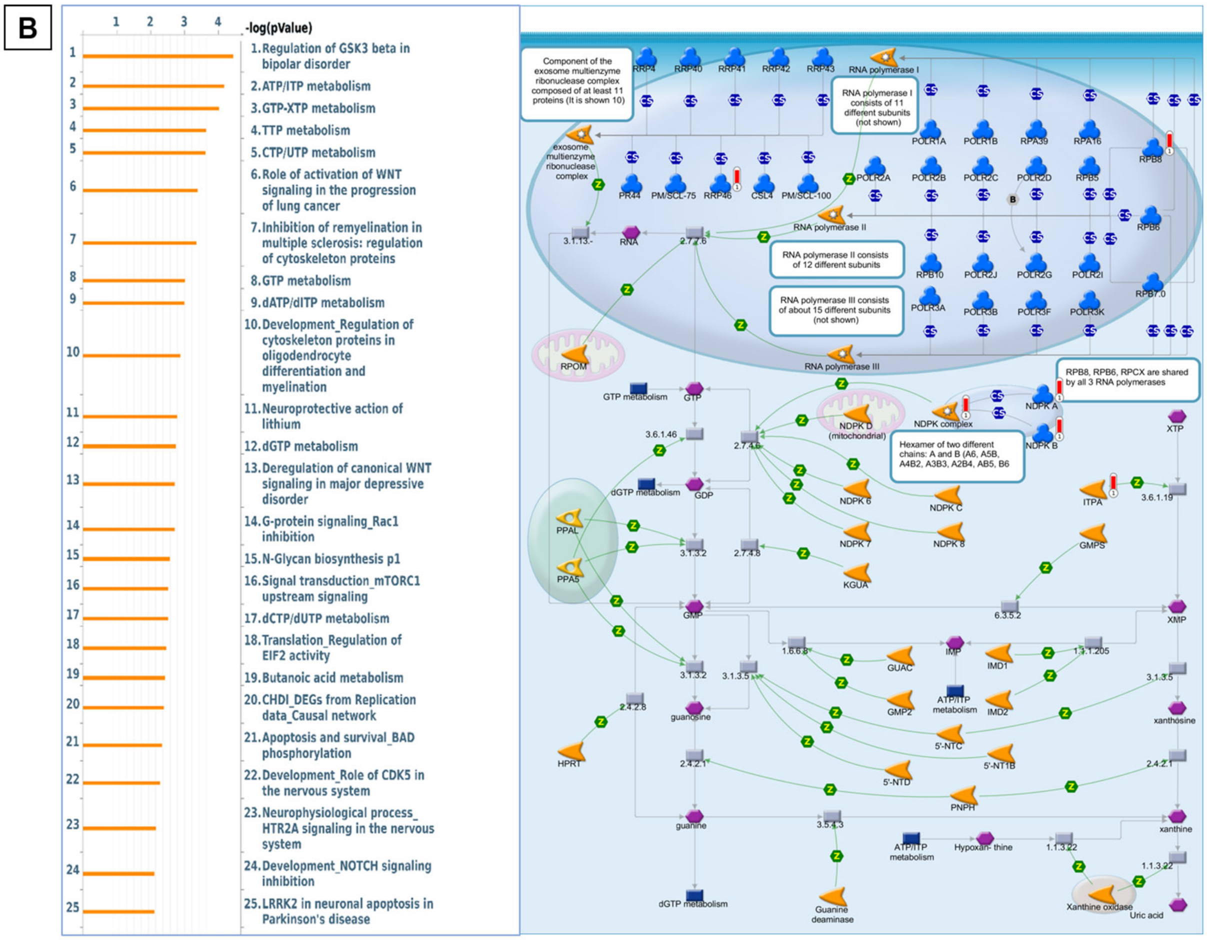


**Supplementary Figure S4. Expression of the DBNDD1 signaling pathway in PCa (MetaCore).** Using the Metacore platform to analyze genes co-expressed with DBNDD1 from the associated TCGA dataset, we found that (**A**) "ATP/ITP metabolism" and (**B**) "GTP-XTP metabolism" were linked to prostate cancer progression (with $p < 0.05$ set as the cutoff value).

## Supplementary Figure S5

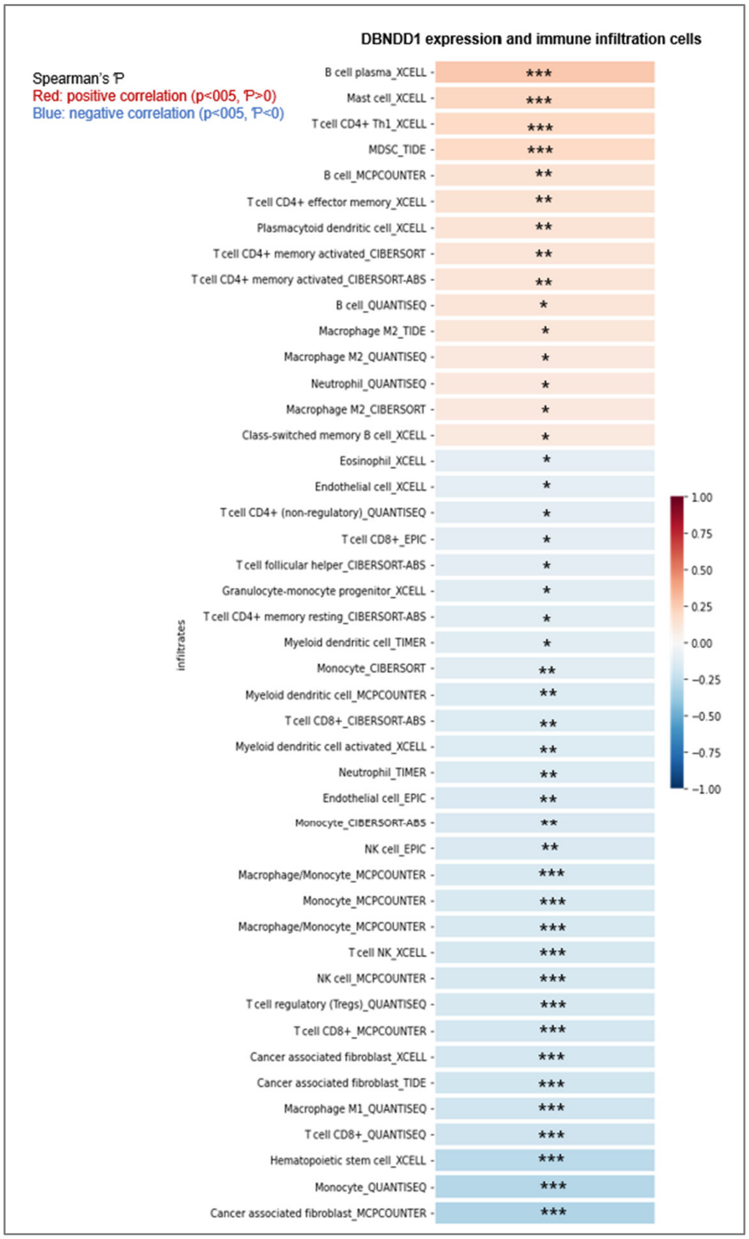


**Supplementary Figure S5. Relationships between DBNDD1 expression and immune cell infiltration in prostate cancer (TIMER).** Correlation coefficients were calculated using cell type quantification algorithms (xCell, Cibersort, Cibersort abs. mode, EPIC, TIDE, MCP-counter, TIMER, and QUANTISED) and are indicated by colors (blue:

negative correlations; red: positive correlations). Statistical significance was defined by $p < 0.05$ (*), $p < 0.01$ (**), $p < 0.001$ (***). Columns: subtypes of prostate cancer. Rows: immune infiltration levels.
